# Transplantation of adipose tissue lacking PAI-1 improves glucose tolerance and attenuates cardiac metabolic abnormalities in high-fat diet-induced obesity

**DOI:** 10.1080/21623945.2020.1748961

**Published:** 2020-04-09

**Authors:** Sijing Liu, Yi Li, Xin Fan, Kai Li, Chunrong Xu, Liping Zhang, Mao Luo, Liqun Wang, Rong Li, Jianbo Wu

**Affiliations:** Key Laboratory of Medical Electrophysiology of Ministry of Education, Collaborative Innovation Center for Prevention and Treatment of Cardiovascular Disease of Sichuan Province, Drug Discovery Research Center, Southwest Medical University, Luzhou, China; Laboratory for Cardiovascular Pharmacology, Department of Pharmacology, School of Pharmacy, Southwest Medical University, Luzhou, Sichuan, China

**Keywords:** PAI-1, transplantation, adipose tissue, glucose, high-fat diet

## Abstract

Adipose tissue is an important metabolic organ, and transplantation of white adipose tissue plays crucial roles in glucose homoeostasis and energy metabolism. However, how adipose tissue affects glucose utilization is poorly understood. PAI-1-knockout (PAI-1KO) mice were previously shown to be resistant to a high-fat diet and obesity. We used microPET/CT (positron emission tomography/computed tomography), gene microarray, and biochemical assays to measure changes in systemic and myocardial glucose metabolism in mice subjected to transplantation of adipose tissue from PAI-1KO and wild-type mice. Here, we show that transplanting subcutaneous white adipose tissue (scWAT) from PAI-1KO mice into high-fat diet (HFD)-fed mice reduced levels of serum total cholesterol and triglycerides, and improved glucose tolerance in the HFD-fed mice. microPET/CT imaging revealed that cardiac glucose uptake was increased in the heart but not in the liver, hindlimb muscles, or abdominal subcutaneous white adipose tissue in HFD-fed mice transplanted with PAI-1KO scWAT, suggesting that the transplanted PAI-1KO scWAT exerted endocrine effects in the heart. In addition, transplantation of scWAT from PAI-1KO mice upregulated mitochondrial gene expression in cardiac muscle, increased the expression of glucose transporters 1 and 4 in cardiac tissues and was associated with an increased NAD^+^/NADH ratio. Together, these findings suggest that modulating PAI-1 in scWAT may provide a promising approach for intervening in glucose metabolism.

## Introduction

Obesity is the primary risk factor for insulin resistance and type 2 diabetes mellitus (T2DM) and determines the extent of complications, such as macrovascular and microvascular diseases and cardiac dysfunction. Abnormalities in adipose tissue (AT) lead to insulin resistance and contribute to the development of obesity and T2DM [1]. Various consequences of AT dysfunction occur and include the accumulation of adipokines such as leptin, adipocyte-specific secretory factor/resistin, and plasminogen activator inhibitor-1 (PAI-1) [2,3]. These adipokines regulate multiple and crucial aspects of whole-body physiology and energy metabolism. Hence, adipose tissue is now recognized as a multi-functional organ that plays an important role in the regulation of energy balance and glucose homoeostasis [4]. White adipose tissue (WAT) is the main energy storage depot and stores energy in the form of lipid within droplets. Indeed, previous studies have demonstrated that changes in the capacity of adipose tissue to synthesize/secrete adipokines are related to the appearance of insulin resistance and T2DM [5,6].

PAI-1 is a crucial component of fibrinolysis and contributes to increased cardiovascular risk in obese and diabetic individuals. In addition, PAI-1 is closely associated with insulin resistance and metabolic risk factor. PAI-1 is mainly produced within white adipose tissue, with adipocytes being the major source of production for circulating PAI-1 in obese individuals. Previous studies have revealed that PAI-1 deficiency protects against obesity and metabolic dysfunction [2,7]. Indeed, our recent study demonstrated that genetic deletion and pharmacological inhibition of PAI-1 improve glucose tolerance and insulin sensitivity [2], which was supported by reduced inflammation and macrophage infiltration into WAT. Further supporting a relationship between PAI-1 and metabolism, an increased release of cytokines has been linked to PAI-1 expression in human adipose tissue explants and obese mice. Collectively, these studies suggest a possible role for AT PAI-1 in the progression of obesity, glucose homoeostasis, and metabolism.

Surgical transplantation of AT is generally believed to have a beneficial effect on obesity, glucose homoeostasis, and insulin sensitivity in high-fat diet-induced obese mouse models [8–10]. In particular, subcutaneous AT implants exhibit an improvement in glucose homoeostasis via endocrine effects in high-fat diet-fed mice [9]. Understanding the detailed mechanisms by which AT transplantation improves glucose homoeostasis is critical for developing potential therapeutic strategies to treat insulin resistance and T2DM. Recently, our results showed that local transplantation of subcutaneous WAT (scWAT) has a pivotal role in improving blood perfusion and systemic metabolism in diabetic limb ischaemia [11]. Although these aforementioned studies provide evidence for a beneficial role of AT transplantation in obesity- and T2DM-related glucose homoeostasis and vascular dysfunction, the underlying mechanisms of increased AT PAI-1 production and its causative relationship with glucose metabolism remain largely unknown.

Based on these findings, in the current study, we tested the hypothesis that AT-derived PAI-1 contributes to the improvement in glucose homoeostasis in HFD-fed model mice. Using a mouse model, transplantation of scWAT from normal chow-fed mice into HFD-fed recipient mice resulted in improved whole-body glucose homoeostasis and cardiac metabolism under conditions of high-fat feeding. These data support a major role for AT-derived PAI-1 in contributing to the metabolic complications of obesity and diabetes.

## Materials and methods

### Animals

C57BL/6 J mice were sourced from the Chongqing Medical University Animal Centre, Chongqing, China. PAI-1-deficient (*Pai1^−/−^*) mice were a gift from Dr. Peter Carmeliet, University of Leuven, Leuven, Belgium [12]. All protocols for animal use were reviewed and approved by the Animal Care Committee of Southwest Medical University in accordance with Institutional Animal Care and Use Committee guidelines.

### HFD-fed mouse model

Eight-week-old male C57BL/6 J mice were fed a HFD (D12451; Research Diet, New Brunswick, NJ) for 14 weeks as described previously [13]. To reduce variation, we used only male mice for all the experiments reported here. Age-matched male mice fed a normal diet (ND) served as controls. Blood glucose levels were measured from tail vein blood samples using an automatic glucometer (Accu-Chek; Roche Diagnostics, Mannheim, Germany). Body weight was monitored every 7 days. Blood was collected and centrifuged at 1500 × *g* for 10 min to measure low-density lipoprotein (LDL), high-density lipoprotein (HDL), total cholesterol (TC), and triglycerides (TG).

#### Adipose tissue transplantation

The transplantation experiments were performed as described previously [14]. Briefly, left inguinal white adipose tissue, weighing ~120 mg, was removed from 8-week-old male normal chow-fed mice and cut into 1- to 2-mm-diameter pieces and transplanted over the deep region between folds within the endogenous epididymal fat of 22-week-old recipient HFD mice fed a high fat diet for 14 weeks. Each recipient HFD-fed mouse received an equivalent transplanted fat mass. Sham surgeries on control mice were performed using the same procedure but without fat pad transplantation. Finally, transplanted mice were sutured, and continued to be fed HFD. Ten weeks after transplantation, glucose metabolism was evaluated. Four to eight mice were used per group over two experiments.

### Micro [^18^ F]FDG PET/CT imaging

Glucose uptake in overnight-fasted mice was determined at 10 weeks post-transplantation by micro [^18^ F]FDG PET/CT images obtained with an Inveon micro PET/CT animal scanner (Siemens, Germany). Mice were anaesthetized with 1% pentobarbital (5 mL/kg), administered an intravenous injection of 100–200 µCi [^18^ F]FDG and positioned in the centre PET field of the view ring. PET/CT images (80 kV; 500 µA; 1.5-mm slice thickness) were acquired 30 min post-[^18^ F]FDG administration. For quantitative analysis, volumes of interest (VOIs) were drawn on PET images for the liver, abdominal subcutaneous white adipose tissue, skeletal muscle (triceps brachii), and heart myocardium. All in vivo images were analysed using PMOD software (PMOD Technologies, Zurich, Switzerland) and Inveon Research Workplace (IRW) software (Siemens Medical Solutions, Knoxville, TN). Four to six mice were used from each group.

### Glucose and insulin tolerance testing

Glucose tolerance tests (GTTs) and insulin tolerance tests (ITTs) were performed using intraperitoneal (IP) injections of D-glucose (Roth, Karlsruhe, Germany) (2 g of glucose/kg total body mass) and insulin (0.75 U insulin/kg total body mass) after a 4-h fast. Blood samples were then obtained from the caudal vein, and the blood glucose level was measured 0, 30, 60, and 120 min after glucose injection using a One Touch® Vita® glucometer (Zug, Switzerland). Four to six mice were used from each group.

### Measurement of plasma PAI-1 and insulin

Blood was collected into citrate anticoagulant and plasma was prepared by centrifugation. PAI-1 was measured using a mouse PAI-1 total antigen assay ELISA kit (Molecular Innovations). Insulin was measured from whole blood by ELISA (Crystal Chem, Downers Grove, IL).

### Microarray

Samples for microarray analysis were prepared following the Affymetrix (Santa Clara, CA) GeneChip Expression Analysis Manual. Total RNA was isolated and purified from frozen cardiac tissues from control or PAI-1KO transplanted mice using TRIzol reagent (Invitrogen) and subjected to cDNA and cRNA preparations. From the total RNA, double-stranded cDNA was created using a SuperScript kit (Invitrogen Life Technologies). The cRNA was synthesized using a MEGAscript T7 Transcription Kit (Life Technologies) and labelled with Cy3-dCTP. The concentration and specific activity of the labelled cRNAs (pmol Cy3/μg cRNA) were measured with a NanoDrop ND-1000 (Thermo Fisher Scientific Inc.). The hybridization solution (40 μL) was dispensed into the gasket slide and assembled to the Agilent SurePrint G3 Mouse GE V2.0 (8x60 K, Design ID: 074809) (Agilent Technologies). The hybridized array was immediately scanned with an Agilent Scanner G2505 C (Agilent Technologies).

The captured images were analysed using Agilent Feature Extraction Software (version 10.7.1.1, Agilent Technologies). Selected gProcessedSignal values were logarithmically transformed and normalized by the quantile method (GeneSpring, version 13.1, Agilent Technologies). The statistical significance of the expression data was determined using fold change. Gene Ontology (GO) functional enrichment analysis for the differentially expressed genes was performed using Gene Set Enrichment Analysis software (http://software.broadinstitute.org/gsea/index.jsp). The gene sets were separated according to the GO terms for biological processes, cellular components, and molecular functions. Pathway analysis was performed using GeneCodis tools (http://genecodis.cnb.csic.es) based on the Kyoto Encyclopaedia of Genes and Genomes (KEGG: https://www.kegg.jp) pathway database. All data analysis and visualization of differentially expressed genes were conducted using R 3.0.2 (www.r-project.org).

### Immunoblotting

Cardiac muscle tissue was homogenized in RIPA buffer (Sigma). The total protein concentration of the homogenates was measured with the BCA reagent. Equal amounts of protein were subjected to SDS-PAGE and transferred to polyvinylidene difluoride membranes by electroblotting. After blocking, the membranes were incubated with antibodies directed against GLUT1 (#ab652; Abcam), GLUT4 (#ab654; Abcam), PAI-1 (#ab222754; Abcam), and GAPDH (#ab9485; Abcam).

### Immunohistochemistry

Cross-sections (5 μm in thickness) were mounted on glass slides, deparaffinized, hydrated, incubated for 10 min in methanol containing 3% H_2_O_2_, and rinsed. PAI-1 immunostaining in WT was performed with a rat anti-mouse PAI-1 mAb (#ab66705; Abcam) and an HRP-conjugated broad-spectrum secondary antibody (Histomouse-MAX Kit; Invitrogen, Carlsbad, CA, USA). As a negative control, immunostaining was also performed by substituting non-immune rat IgG for anti-PAI-1 IgG. The images were captured using a microscope (Leica, Germany). The numbers were quantified in 5 microscopic fields in each of the 3 cross-sections of each tissue using ImagePro Plus software.

### NADH and NAD^+^/NADH ratio measurements

Homogenates from mouse *cardiac muscle* at 10 weeks post-transplantation were used to analyse the NAD^+^/NADH ratio. The total intracellular NAD^+^/NADH ratio was measured using a CheKine™ NAD^+^/NADH Assay Kit (Abbkine Inc., China).

### Statistical analysis

Data are presented as the mean ± SEM. Experimental groups were compared by non-parametric, two-tailed Mann-Whitney or one-way analysis of variance (ANOVA). A level of *P* < 0.05 was defined as indicative of statistical significance.

## Results

### Transplantation of scWAT from PAI-1KO mice improves glucose homoeostasis

**Figure 1.**
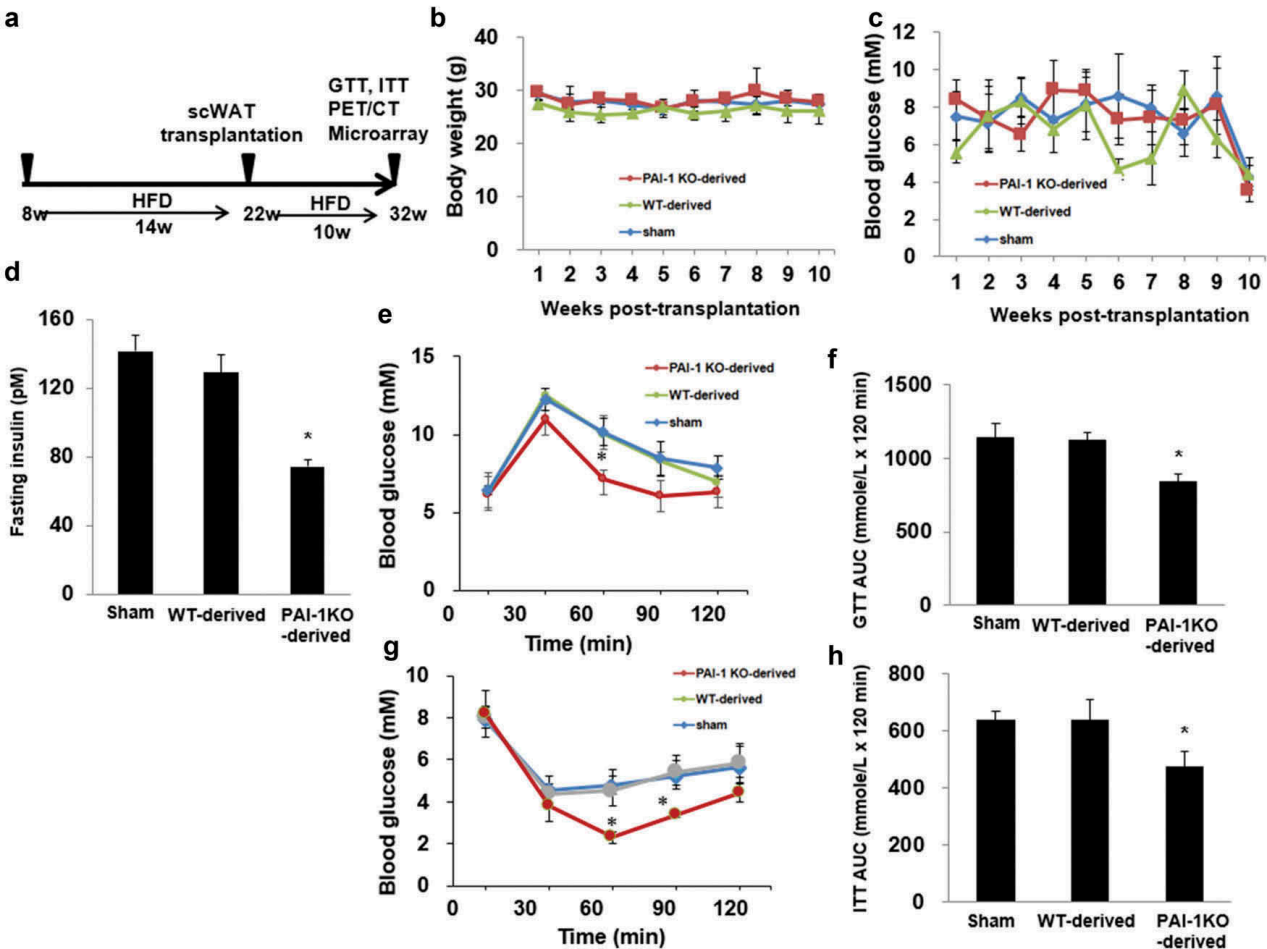
**Transplantation of subcutaneous adipose tissue from PAI-1KO mice improves glucose tolerance and insulin sensitivity**. (a) Schematic timeline of scWAT transplantation model. (b) Body weight and fasting glucose level (c) in each group. (d) Insulin levels were measured by ELISA in each group. n = 4–6 per group. *P < 0.05 vs. WT transplanted and sham. (e, f) Glucose tolerance tests (GTTs) and AUC (area under the curve) in each group. n = 4–6 per group. *P < 0.05 vs. WT transplanted and sham. (g, h) Insulin tolerance tests (ITTs) and AUC in each group. n = 4–6 per group. Results are expressed as means ± SEM. Differences between experimental groups were analysed by one-way ANOVA. Significance of differences was set at *P* < 0.05. *P < 0.05 vs. WT transplanted and sham

Transplantation of scWAT from normal and PAI-1KO mice was not associated with changes in body weight or fasting glucose (C). Mice transplanted with scWAT from WT and PAI-1KO mice exhibited a decrease in cholesterol levels 10 weeks post-transplantation compared with sham-treated controls (Table 1). This agrees with our previous findings that transplantation of WAT led to significantly lower total plasma and HDL cholesterol concentrations in recipient HFD-fed mice [11]. No changes in plasma PAI-1 levels were observed between groups. The expression of PAI-1 in transplanted depot and surrounding WAT was significantly reduced in recipient HFD-fed mice receiving scWAT from PAI-1KO mice compared with sham (Supplementary Figure 1(a-d)). By 10 weeks after transplantation, the insulin levels were significantly reduced in recipient HFD-fed mice receiving scWAT from PAI-1KO mice compared with both the sham and control mice (Figure 1(d)), Furthermore, an intraperitoneal glucose tolerance test (IGTT) was performed in a separate cohort of mice at 10 weeks post-transplantation. Consistent with our previous results [10], mice transplanted with scWAT from PAI-1KO mice showed a significant improvement in glucose tolerance compared with both sham-treated mice and mice receiving normal scWAT (*P* < 0.01) (Figure 1(e-f)). Interestingly, there was no difference in glucose tolerance between mice receiving scWAT from C57BL/6 J mice and sham-treated controls 10 weeks post-transplantation. Thus, transplantation of scWAT from PAI-1KO mice into the viscera improves glucose tolerance.

**Table 1.** Plasma lipid profiles

Group	TC	TG	LDL-C	HDL-C
Sham	3.31 ± 0.22	0.3 ± 0.06	0.57 ± 0.03	1.98 ± 0.11
Transplanted WT	2.89 ± 0.29	0.45 ± 0.05	0.36 ± 0.42^#^	1.30 ± 0.17^#^
Transplanted PAI-1KO	2.77 ± 0.31^#^	0.34 ± 0.09	0.33 ± 0.06^#^	1.59 ± 0.15^#^

All units are mM. Values are mean±SEM. ^#^^*P*^< 0.05 *vs*. sham fed western diet; n = 6 mice per group. TC, total cholesterol; TG, triglycerides; LDL-C, low-density lipoprotein cholesterol; HDL-C, high-density lipoprotein cholesterol.

Furthermore, mice transplanted with scWAT from PAI-1KO mice had a greater insulin-induced decrease in glucose levels than both sham-operated mice and mice transplanted with scWAT from normal mice (Figure 1(g-h)), revealing increased peripheral insulin sensitivity.

### Transplantation of scWAT from PAI-1KO mice increases glucose uptake in cardiac muscle

**Figure 2.**
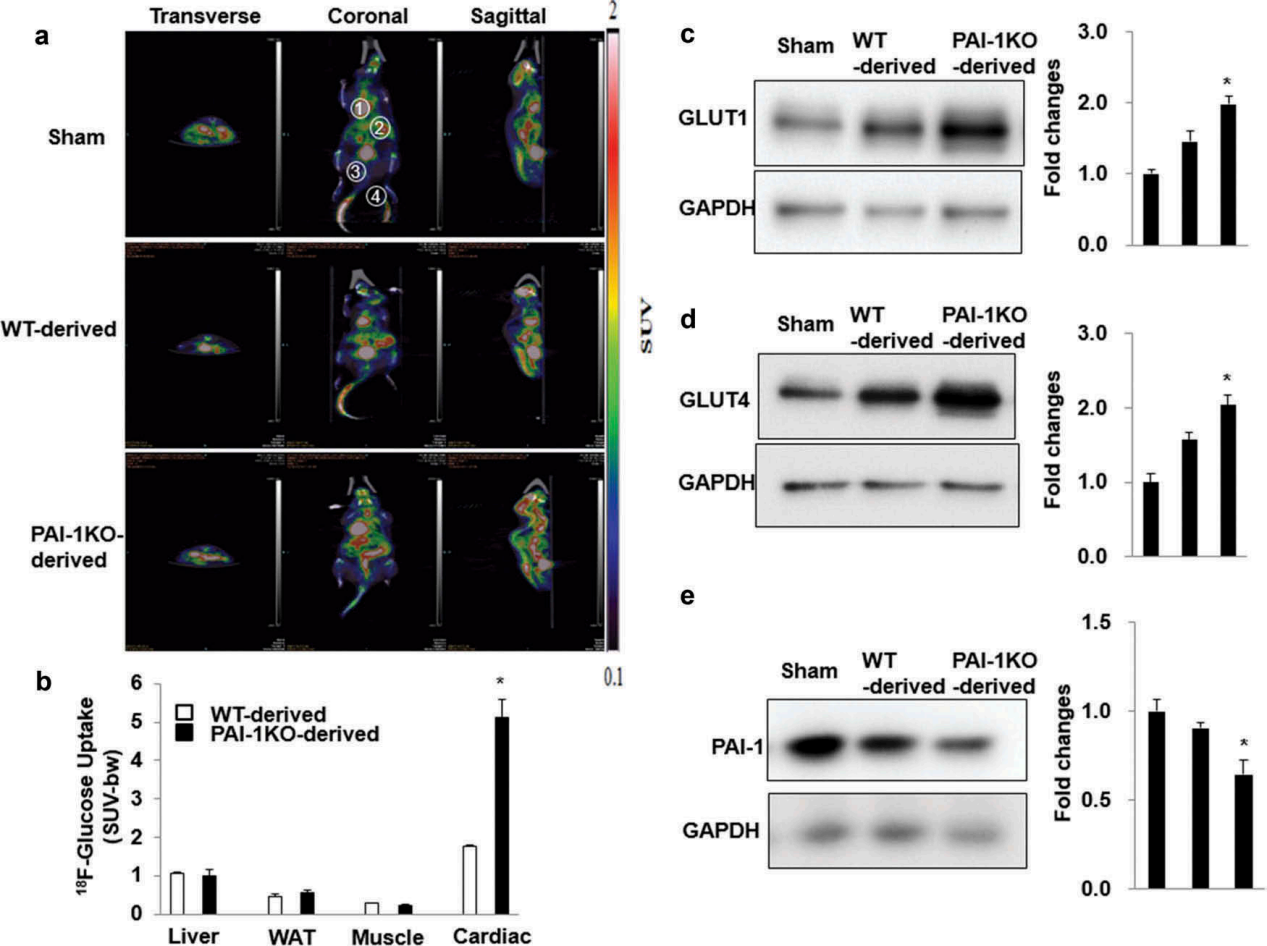
**Transplantation of scWAT from PAI-1KO mice increases glucose uptake in cardiac muscle**. (a) Transverse, coronal, and sagittal PET/CT images of mice through the liver, abdominal subcutaneous white adipose tissue, gastrocnemius muscle, and heart. (b) PET intensities were normalized to total positron emissions for each mouse and plotted against time. The liver, WAT, gastrocnemius muscle, and heart are presented. Bars are the means±SEM, *p* values were generated with ANOVA; n = 4-6/each group. (c-e). Cardiac tissue lysates were prepared and subjected to western blotting to detect GLUT1 (c), GLUT4 (d), and PAI-1 (e). Representative images of 3 independent experiments are shown. All graphs correspond to the blots above them and represent densitometric analyses of 3 independent experiments. Differences between experimental groups were analysed by one-way ANOVA.*P < 0.05 vs sham and WT-derived

To evaluate the organs and tissues responsible for the improvement in glucose tolerance 10 weeks post-transplantation in mice receiving scWAT from PAI-1KO mice, we performed small animal PET/CT imaging on a subset of the mice using [^18^ F]FDG as a tracer. Basal (non-insulin–stimulated) [^18^ F]FDG uptake in tissues such as the liver, abdominal scWAT, skeletal muscle, and cardiac muscle was analysed, and the results showed that basal cardiac muscle glucose uptake in the PAI-1KO-derived WAT group was significantly increased 30 min after the injection of [^18^ F]FDG compared to that in either the sham or control WAT group (Figure 2(a,b)). However, glucose uptake in the liver, scWAT, and skeletal muscle was similar in the three groups (Figure 2(a-b)). Thus, in the absence of exogenous insulin under fasting conditions, transplantation of scWAT from PAI-1KO mice into HFD-fed mice results in increased glucose uptake into cardiac muscle.

We further determined the expression of glucose transporters in cardiac muscle by using western blotting analysis. Consistently, transplantation of scWAT from PAI-1KO mice increased GLUT4 and GLUT1 expression in the cardiac muscle of recipient mice compared with that in both the sham and control mice (Figure 2(c-e)). Interestingly, cardiac expression of PAI-1 protein was significantly reduced in recipient HFD-fed mice receiving scWAT from PAI-1KO mice compared with both the sham and control mice. Altogether, our results demonstrated that transplantation of scWAT from PAI-1KO mice increased glucose utilization in HFD-fed mice, likely by enhancing glucose uptake by cardiac muscle via induction of GLUT1 and GLUT4.

### Transplanted scWAT from PAI-1KO mice upregulated mitochondrial gene expression and function in cardiac muscle

**Figure 3.**
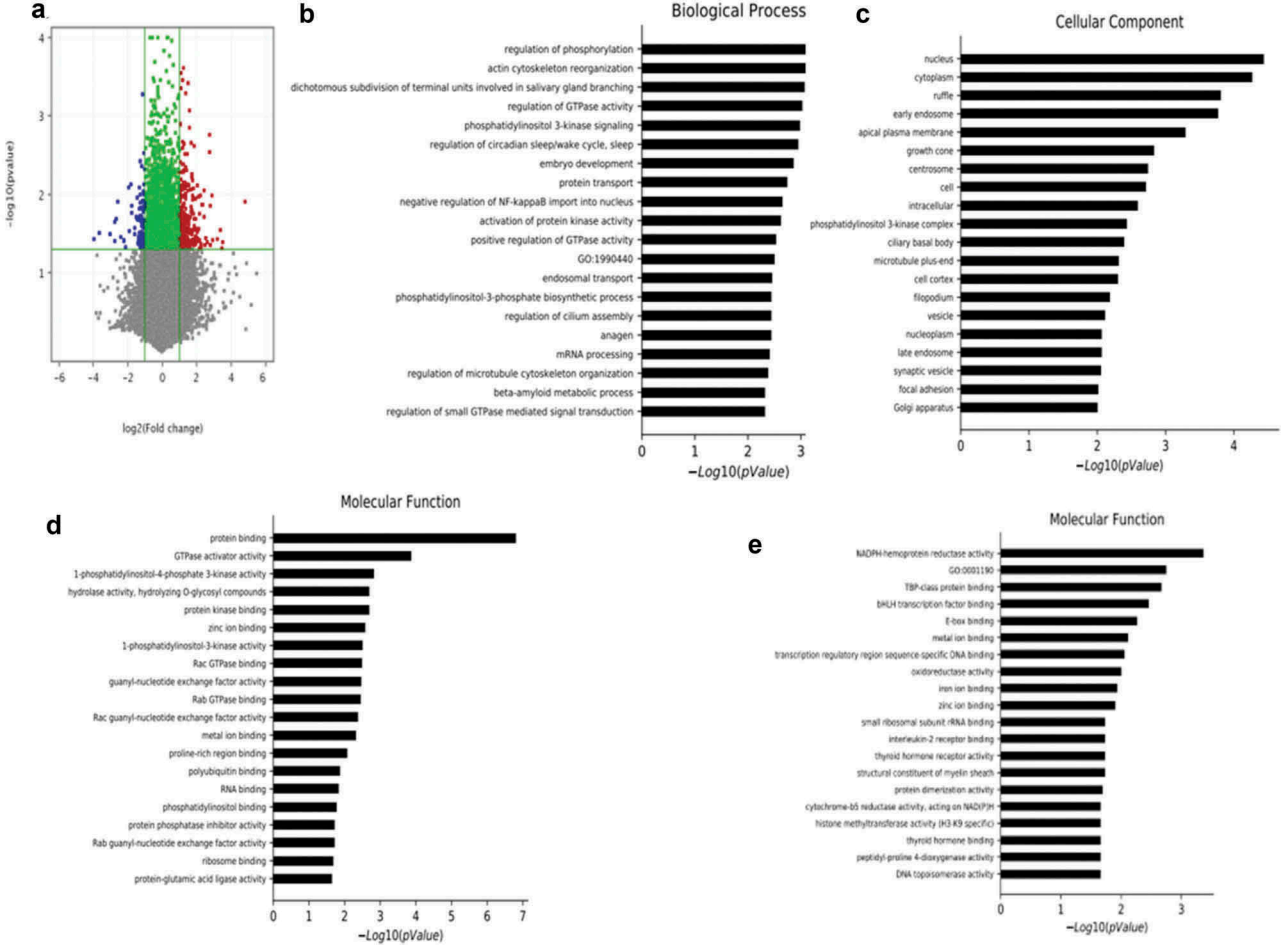
**Transplantation of scWAT from PAI-1KO mice leads to alterations in cardiac muscle mRNA**. (a) Volcano plot showing the differentially expressed genes in cardiac muscle due to transplanted PAI-1KO and WT scWAT. The negative log_10_-transformed *p* values are plotted against the average log_2_ fold changes in gene expression. Data for genes that were not classified as differentially expressed are plotted in grey (*p* > 0.05). Data for genes that are differentially expressed due to transplanted PAI-1KO scWAT (*p* < 0.05) with an absolute log_2_ fold change of less than 2 are plotted in green. Data for genes that are differentially expressed due to transplanted PAI-1KO scWAT (*p* ≤ 0.05) with an absolute log_2_ fold change of greater than or equal to 2 are plotted in red, and those with an absolute log_2_ fold change of less than or equal to −2 are plotted in blue. (b-d). The significantly upregulated GO terms of mitochondrial function for differentially expressed mRNAs, including biological process (b), cellular component (c), and molecular function (d). (e). The significantly downregulated GO terms of fatty acid oxidation for differentially expressed mRNAs. GO, gene ontology

To gain insight into the gene expression changes in cardiac muscle in transplanted mice, we performed microarray analysis. As shown in Dataset S1, differentially expressed mRNAs were identified between mice receiving scWAT from the PAI-1KO and WT groups. Using a 2-fold expression difference as a cut-off, 585 differentially expressed mRNAs (400 upregulated and 185 downregulated) were identified between the two groups (Figure 3(a)). The GO analysis is used to categorize and describe the biological functions of genes, covering three domains: biological process, cellular component and molecular function. According to GO analysis, upregulated genes were mainly involved in mitochondrial function, including biological process (Figure 3(b)), cellular component (Figure 3(c)) and molecular function (Figure 3(d)). Conversely, fatty acid oxidation was downregulated (Figure 3(e)).

**Figure 4.**
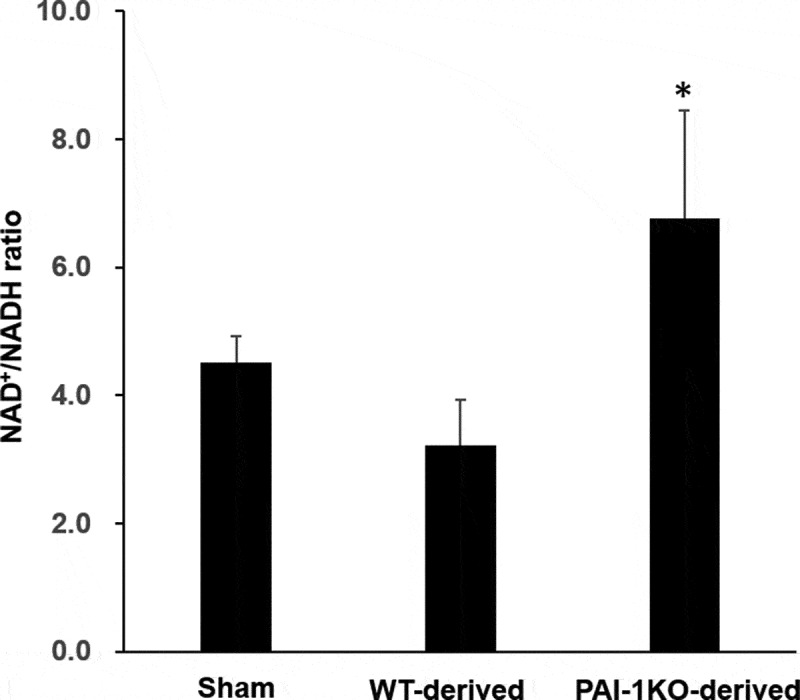
**Transplantation of scWAT from PAI-1KO mice increases the NAD^+^/NADH ratio in cardiac muscle**. The total intracellular NAD^+^/NADH ratio of heart tissues was measured from sham, WT, and PAI-1KO transplanted mice. Bars are the means±SEM; n = 6 mice per group. *P < 0.05 vs. WT transplanted and sham

Since the NAD/NADH ratio is required for efficient mitochondrial metabolism, we next measured the NAD^+^/NADH ratio in cardiac tissues obtained from sham, normal, and PAI-1KO transplanted mice. The results showed that mice transplanted with PAI-1KO scWAT exhibited a significantly increased NAD^+^/NADH ratio in cardiac tissues compared with sham mice and mice transplanted with normal scWAT (Figure 4), suggesting that transplantation with PAI-1KO scWAT improved cardiac mitochondrial function.

### Discussion

In the present study, we demonstrated that transplantation of scWAT from PAI-1KO mice improves glucose tolerance and insulin sensitivity compared with sham-transplantation or transplantation of scWAT from WT mice, and the process of transplantation also stimulates glucose utilization by enhancing glucose uptake in cardiac muscle. Thus, PAI-1, a major adipokine, is involved in exerting metabolic effects through AT transplantation.

As recently demonstrated by us, inhibition of PAI-1 exerts beneficial effects on energy and glucose metabolism; moreover, PAI-1 contributes to the development of inflammation in adipose tissue and explains the mechanism of PAI-1-modulated inflammation in the disordered metabolism in HFD-induced obesity [1]. Excessive tissue accumulation of PAI-1 has been shown to cause vascular dysfunction, and paradoxically, genetically deficient PAI-1 protects against cardiac fibrosis [15], suggesting that PAI-1 exerts a fundamentally unique role in cardiac tissues. Previous studies have shown that increased cardiac PAI-1 expression is associated with insulin resistance in genetically obese mice [16]. In the present study, we found that transplantation of scWAT from PAI-1KO mice induced an inhibitory effect on PAI-1 expression in cardiac tissues of HFD mice, suggesting that PAI-1 and other unknown factors produced from transplanted AT impact on a wide range of physiological processes and target tissues. The specific mechanisms of scWAT involved in decreasing the PAI-1 level in cardiac tissue need further study.

A key finding from the current study was the marked improvement in systemic glucose homoeostasis and glucose uptake in the heart observed after transplantation of scWAT from PAI-1KO mice. The effects on glucose metabolism in circulation and target tissues must involve metabolic benefits conferred by the transplanted WAT, and glucose regulation may be maintained completely independent of the endocrine pancreas. WAT is a versatile endocrine organ that secretes a range of hormones that influence physiological and pathophysiological functions related to metabolism and the inflammatory response [11,17]. Previous studies have shown that transplantation of healthy adipose tissue can compensate for the function of the endocrine pancreas [6,8]. Accordingly, our data indicate that AT-derived PAI-1 is important in glucose hemostais but the role of endocrine signalling from the transplanted AT deserves further investigation.

Moreover, the transplantation of scWAT from PAI-1KO mice resulted in increased glucose uptake in the cardiac muscle of recipient mice.

Microarray data of cardiac muscle revealed that a vast number of genes involved in many cellular functions were significantly upregulated or downregulated following the transplantation of scWAT from PAI-1KO mice. A number of the genes that were upregulated by transplanted PAI-1KO scWAT had significant effects on cardiac tissue genes involved in mitochondrial biogenesis and downregulation of fatty acid oxidation. In genetic mouse models of T2DM and human obesity, there are marked increases in myocardial fatty acid oxidation and decreases in glucose oxidation rates [18,19]. These changes in myocardial energy metabolism are associated with a decline in myocardial glucose uptake in T2DM patients with diastolic dysfunction [20,21]. Cardiac dysfunction is characterized by a state of metabolic abnormalities involving enhanced rates of fatty acid uptake and mitochondrial dysfunction. Intracellular NAD^+^/NADH is mainly localized within mitochondria [22]. The NAD^+^/NADH ratio indicates the activity of various metabolic pathway enzymes, such as those involved in mitochondrial oxidation as the predominant energy source. Notably, a previous study demonstrated that a decreased NAD^+^/NADH ratio was associated with diabetic cardiomyopathy [23]. Our results revealed that mice transplanted with PAI-1KO scWAT had a significantly increased NAD^+^/NADH ratio in cardiac tissues compared with that in the WT and sham groups, suggesting that targeting PAI-1 is necessary for preventing the metabolic complications caused by obesity and T2DM.

Evidence from mouse models demonstrates that glucose uptake plays a crucial role in myocardial protection. Our investigation into this metabolic profile revealed that myocardial glucose uptake in association with cardiac GLUT1 and GLUT4 expression was significantly increased in the transplantation of scWAT from PAI-1KO mice. GLUT1 and GLUT4 have previously been identified as targets for transcriptional regulation in adipose tissue and skeletal muscle. GLUT4 is strongly expressed in cardiac tissue for glucose uptake, but in diabetic patients, the decrease in GLUT4 expression has been correlated with a decrease in the uptake and utilization of glucose [24]. The mechanism for this effect is not clear but may involve mitochondrial function and is believed to be an adaptive response that confers cardioprotection [25,26].

In summary, we provided evidence showing that transplantation of scWAT from PAI-1KO mice is a key regulator of glucose utilization and homoeostasis. Our results suggest that targeting PAI-1 is a promising approach for the treatment of diabetes and its complications.

## Supplementary Material

Supplemental Material
