# Supplementary figures and images for: Transplantation of adipose tissue lacking PAI-1 improves glucose tolerance and attenuates cardiac metabolic abnormalities in high-fat diet-induced obesity
Source: Adipocyte. 2020 Apr 9;9(1):170–8. doi: 10.1080/21623945.2020.1748961 (PMC7153656; doi:10.1080/21623945.2020.1748961)

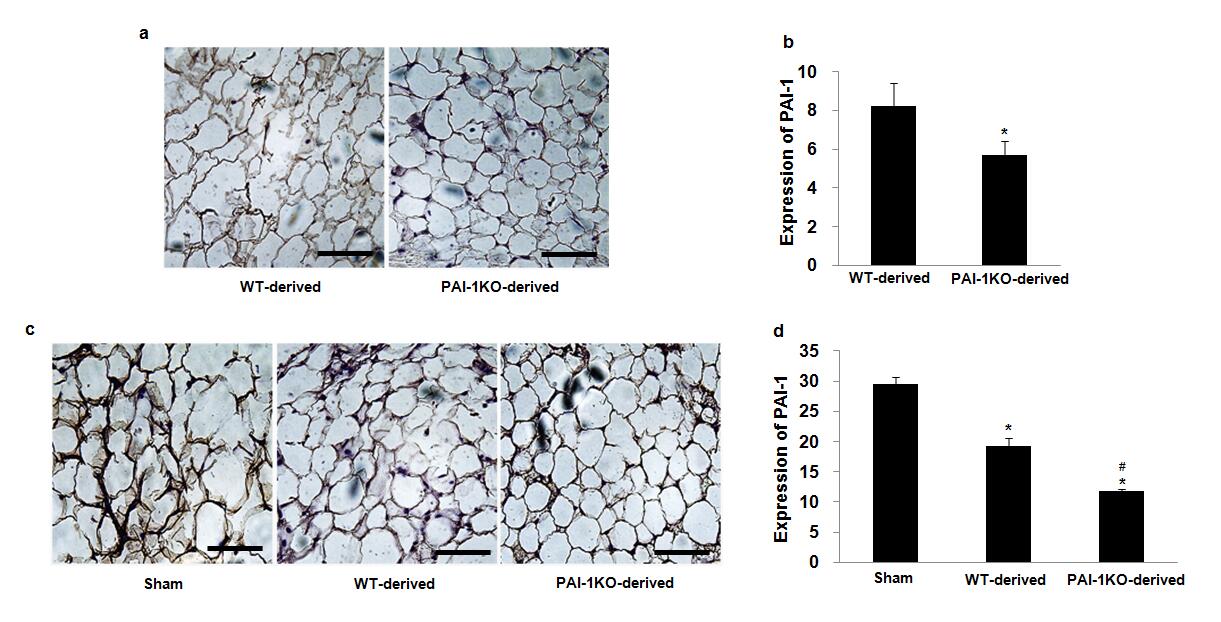

Supplement: Supplemental Material [file KADI_A_1748961_SM7617.jpg]
